# Regulatory Crosstalk of Doxorubicin, Estradiol and TNFα Combined Treatment in Breast Cancer-derived Cell Lines

**DOI:** 10.1038/s41598-019-51349-9

**Published:** 2019-10-23

**Authors:** Isar Nassiri, Alberto Inga, Erna Marija Meškytė, Federica Alessandrini, Yari Ciribilli, Corrado Priami

**Affiliations:** 1 Department of Oncology, MRC Weatherall Institute of Molecular Medicine, University of Oxford, Oxford, UK; 2 The Microsoft Research – University of Trento Centre for Computational and Systems Biology (COSBI), Rovereto, TN Italy; 3 Laboratory of Transcriptional Networks, Department CIBIO, University of Trento, 38123 Trento, Italy; 4 Laboratory of Molecular Cancer Genetics, Department CIBIO, University of Trento, 38123 Trento, Italy; 5 Department of Biological Models, Life Sciences Centre, Institute of Biochemistry, Vilnius University, Vilnius, Lithuania; 6 Dipartimento di Informatica, Università di Pisa, Pisa, Italy

**Keywords:** Cancer models, Cellular signalling networks

## Abstract

We present a new model of ESR1 network regulation based on analysis of Doxorubicin, Estradiol, and TNFα combination treatment in MCF-7. We used Doxorubicin as a therapeutic agent, TNFα as marker and mediator of an inflammatory microenvironment and 17β-Estradiol (E2) as an agonist of Estrogen Receptors, known predisposing factor for hormone-driven breast cancer, whose pharmacological inhibition reduces the risk of breast cancer recurrence. Based on the results of transcriptomics analysis, we found 71 differentially expressed genes that are specific for the combination treatment with Doxorubicin + Estradiol + TNFα in comparison with single or double treatments. The responsiveness to the triple treatment was examined for seven genes by qPCR, of which six were validated, and then extended to four additional cell lines differing for p53 and/or ER status. The results of differential regulation enrichment analysis highlight the role of the ESR1 network that included 36 of 71 specific differentially expressed genes. We propose that the combined activation of p53 and NF-kB transcription factors significantly influences ligand-dependent, ER-driven transcriptional responses, also of the ESR1 gene itself. These results provide a model of coordinated interaction of TFs to explain the Doxorubicin, E2 and TNFα induced repression mechanisms.

## Introduction

Cancer cells undergo uncontrolled proliferation and genome instability due to the induction or suppression of regulatory mechanisms of normal cells^1^. Studying gene expression profiles resulting from interactions between signal-activated transcription factors is an efficient method for better understanding the complex, unstable and adapted gene regulatory networks system of cancer cells^2^. In this study, we compared the transcriptomics of MCF-7 cells under the combined treatment with Doxorubicin, 17β-Estradiol (E2) and TNFα with single or double treatments. Doxorubicin, a first line chemotherapeutic agent, promotes the activity of p53 through DNA damage response, which is associated with a combination of cell cycle arrest and the programmed cell death^3,4^. Estradiol is an agonist ligand of the Estrogen Receptors (ER, primarily ERα coded by the ESR1 gene, but also ERβ coded by the ESR2 gene, and the more elusive Estrogen-related Receptors ERRs), and promotes the proliferation of cancer cells^3^. The increased expression of estrogen and progesterone receptors in cancer cells can promote the growth and survival of tumor cells. Hyperactivity of ER can reduce the effectiveness of regulatory mechanisms for repression of ESR1 expression as a predisposing factor for hormone-driven breast cancer^5–8^. TNFα is an important inflammatory cytokine that can mediate both cytotoxic and growth promoting effects in breast cancer^9,10^. Some studies showed that double treatment of MCF-7 with TNFα and E2 had antagonistic effects on cell proliferation and survival^11,12^. Although the molecular mechanism of the inhibitory effect of TNFα on the estrogen-induced proliferation of MCF-7 is not well understood, two published studies have provided some clues: (1) the down-regulation of ESR1 through the PI3K/AKT signalling was introduced as a mechanism of suppression of E2-mediated cell proliferation by TNFα in MCF-7^11^; (2) p53 wild-type and p53-mutated MCF-7 cells respond differently to estrogen and TNFα treatment^1^. However, reports vary, as TNFα and other cytokines were shown to activate ER, independently from the presence of the E2 ligand^13,14^. For instance, TNFα effects on the ER by activation of adaptor protein TAB2 and NCoR/SMRT corepressors to ER, and induces loss of the antiproliferative responses^15^. In previous studies, we analyzed the interaction between differentially regulated pathways in response to the double treatments of MCF-7 with Doxorubicin + TNFα, DT) or Doxorubicin + E2 (DE), and reported on the identification of additive and synergistic gene expression modulation^2,16,17^. In this study, we focus on the combined triple treatment Doxorubicin + TNFα + E2 (DTE) in comparison with single or double treatments. We used network-based functional analysis of transcriptomic profiles to identify the cellular impacts of combinatorial treatments that are expected to result in the activation of p53, ER, and NF-kB transcription factors. Functional analysis of transcriptomics data is usually performed by enriching differentially expressed genes (DEGs) to the predefined reference gene sets as indicators of different biological processes^18^. To understand the regulatory mechanisms that operate changes in gene expression, gene regulatory inference methods construct networks based on the regulatory interaction between the DEGs^19^. Other algorithms use prior knowledge about the interaction between molecules, for instance, based on PPI networks^20^. We developed a tool called *NASFinder* for topological and functional analysis of networks associated with omics data^21^. *NASFinder* follows a two-step procedure: (1) it tests an entire generic network to identify significant sub-networks with their associated topology and transcription factors that correlate with the expression of DEGs; (2) it functionally analyses the identified sub-networks and ranks them in term of the magnitude of the expression fold changes by using the network activity score index (NAS – see the Methods section for a precise definition). We used this method to understand the regulatory mechanisms that operate changes in gene expression due to the simultaneous combination treatments leading to activation of p53, ER and NF-kB transcription factors in the MCF-7 breast cancer cell line.

## Results

The main result of this study is a new model of ESR1 network repression as an exclusive gene expression program of MCF-7 responding to the combinatorial treatment with Doxorubicin, TNFα and E2. Furthermore, we studied the key regulatory molecules and modules in an integrated network of differentially expressed genes, as potential targets for cancer therapy. The experimental setup and transcriptomic data were presented in detail in our two previous studies focusing on the crosstalk between Doxorubicin + E2 or between Doxorubicin + TNFα^2,17^.

### Biological network pathways associated with Doxorubicin, Estradiol and TNFα treatments

**Figure 1:**
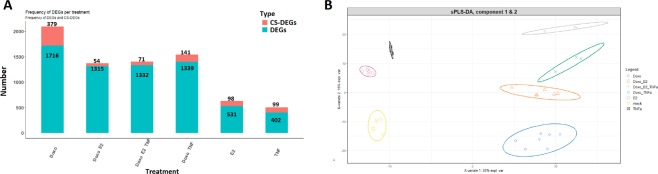
The distribution of differentially expressed genes (DEGs) and context-specific differentially expressed genes (cs-DEGs) in six studies associated with treatments of MCF-7 breast cancer cell line. (**A**) The plot represents the frequencies of DEGs and CS-DEGs in response to the treatments. The number of CS-DEGs was extracted based on the pairwise comparison of Doxorubicin + E2 (10^−9^ M), Doxorubicin + E2 (10^−9^ M) + TNFα, Doxorubicin + TNFα, Doxorubicin, and E2 (10^−9^ M). (**B**) Clustering of samples using PLS-DA of the expression matrix of DEGs. Confidence ellipses for each cluster highlight the strength of the discrimination (confidence level 95.4%).

**Figure 2:**
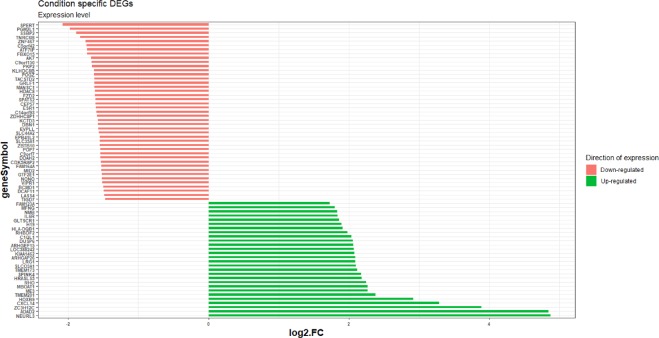
The list and expression patterns of context-specific DEGs in transcriptomics profile of MCF-7 cell line in response to the treatment with Doxo + TNFα  + E2 in comparison to the single and double treatments. The minus value of fold change means down-regulated and positive value indicates up-regulated.

MCF-7 cells were treated with 1.5 μM Doxorubicin, 10^−9^ *M* E2 and 5 ng/ml TNFα and total RNA were extracted 10 hours post-treatment. Global gene expression analyses were conducted as described in^17^. All datasets have been deposited in GEO (GSE24065 and GSE50650). Screening differentially expressed genes across 6 conditions (single and combination treatments with Doxorubicin, Estradiol, and TNFα) indicated exclusive DEGs in different conditions, called context-specific DEGs (CS-DEGs) (Figs 1A and 2) (Supplementary File 1). The number and expression patterns (i.e. up- or down-regulated) of differentially expressed genes among data sets were heterogeneous (Supplementary File 1). The supervised multivariate analysis (PLS-DA) was performed in order to cluster groups of DEGs. The PLS-DA model was fitted with six components, and samples were projected onto subspaces spanned by the first two components. From the PLS-DA plot, we observe a clear separation of the single, double, and triplet treatments (Fig. 1B). These results indicated that the expression patterns of the DEGs in response to the different treatments were distinct. Thus, it was speculated that CS-DEGs might be considered as key regulatory molecules in response to the Doxorubicin, TNFα, and estrogen exposure.

### Network-based gene set enrichment analysis of context-specific DEGs

**Figure 3:**
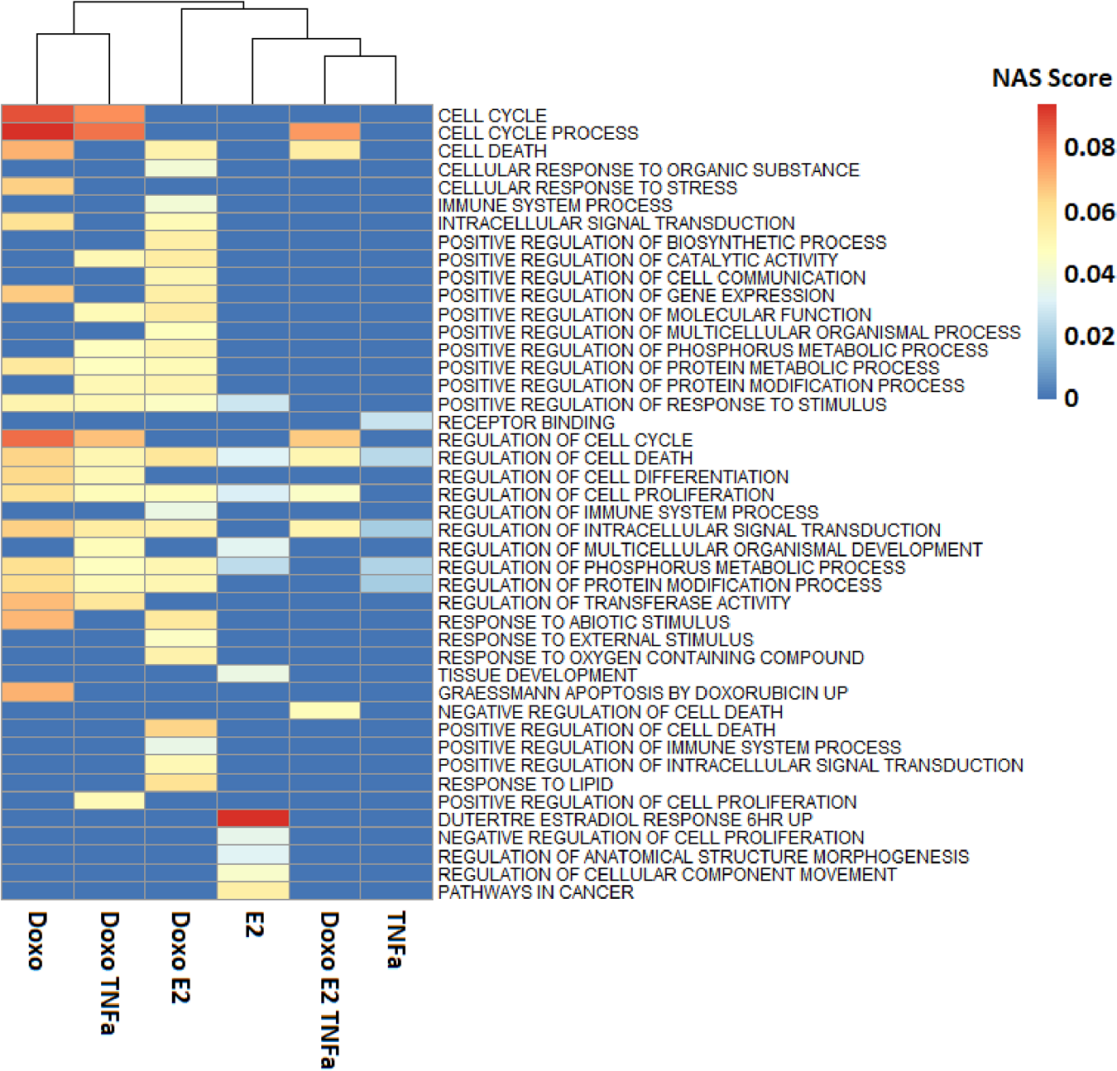
Active networks in response to varying treatments with chemotherapeutic agents. Enriched pathways obtained through the gene set enrichment analysis of the context-specific DEGs for each data set. Pathway annotations of context-specific DEGs were obtained from *NASFinder*. Color scales are proportional to the network activity score (NAS) values. NAS considers the impact of selected cellular processes in the experimental context. Doxo and E2 are abbreviations of Doxorubicin and 17β-Estradiol 10^−9^ *M*, respectively (Supplementary File 2).

For a network analysis of the impact of the triple treatment, the list of context-specific DEGs were used for identification of active network pathways under the regulatory control of receptors. The active networks were ranked according to the network activity scores (NAS) and the 44 top cases of combined treatment with a significant p-value (<0.0001) were compared with single or double treatments (Supplementary File 2). The details and parameters used for this analysis are described in the Methods section. The CS-DEGs were encoding cytoskeletal, immune system, and transmembrane transport proteins as expected, and represent potential for signal transduction drug response markers in breast cancer therapy. Networks related to cell death, proliferation, and intracellular signal transduction were common among the enriched groups (Fig. 3). Activated networks in response to the TNFα treatment was important in apoptosis under the regulation of androgen receptor (AR), and double treatment of Doxorubicin + TNFα shifted the top results to the signalling response to cell cycle process (Fig. 3). As expected, the regulated networks with high NAS score in response to the treatment with Doxorubicin and Estradiol were *apoptosis by Doxorubicin* and *Estradiol response*, respectively. Doxorubicin + E2 treatment induced related pathways to the *regulation of cell proliferation* (Fig. 3). *NASFinder* identified 26 regulated network pathways (Adjusted P-value ≤0.05) in response to the combined treatment with Doxorubicin, Estradiol, and TNFα mainly related to cell cycle and related processes (Fig. 3) (Supplementary File 2). It was found that ESR1 was regulating the module of context-dependent DEGs in response to the triple treatment (Supplementary File 2). These findings suggested that the CS-DEGs play an important role in the response of breast cancer to the treatments. In the next steps, we combined these topological and functional findings with information about the expression patterns of the context-specific set of DEGs for studying the molecular mechanisms of the impact of Doxorubicin + TNFα  + E2 treatment on cellular processes.

### The ESR1 is a key CS-DEGs in the integrated network of enriched genes

**Figure 4:**
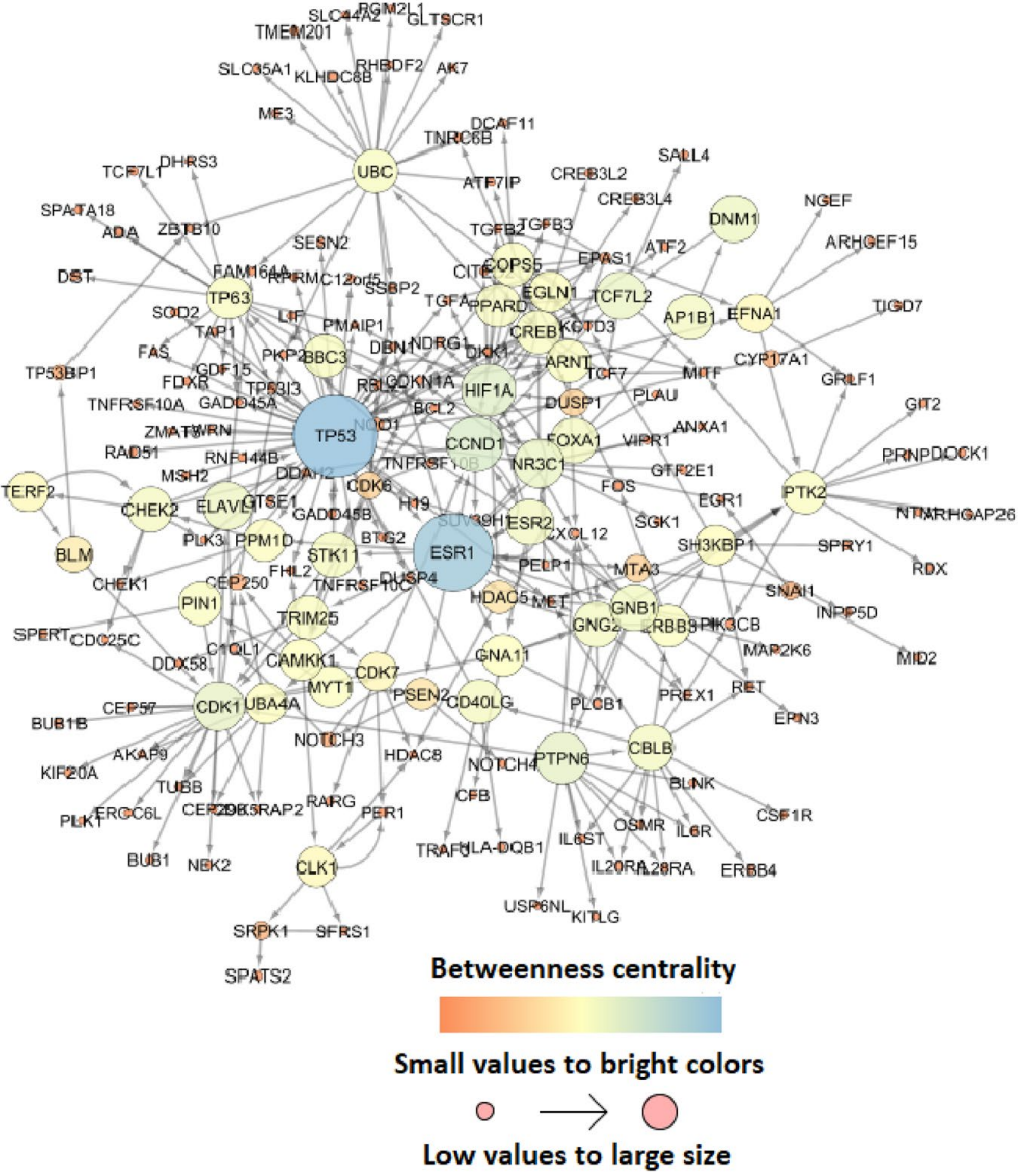
The integrated network of enriched genes related to the combined treatment of MCF-7 with Doxorubicin, TNFα, and E2. The centrality of molecules in information flow through the network is based on the betweenness centrality index. Nodes with low betweenness values have av small and bright color circle. The high brightness colour of edges presents low edge betweenness values. The node degree distribution of the network fits the power law^22^.

**Figure 5:**
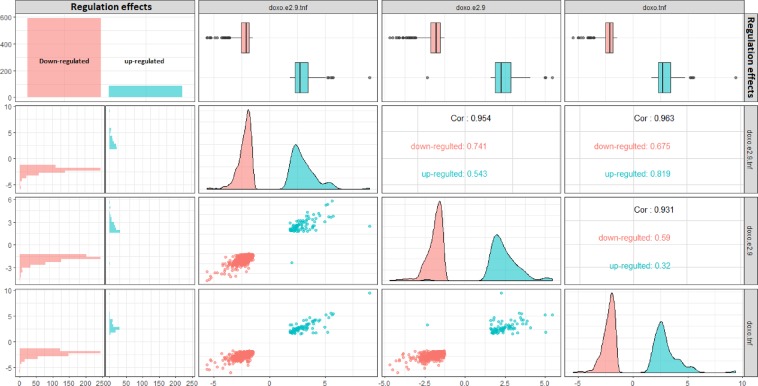
The generalized pairs plot of correlation between the fold changes of common differentially regulated genes in response to the double and triple treatments with Doxorubicin, Estradiol, and TNFα. Down-regulated genes mainly mediate MCF-7 response to Doxorubicin/Estradiol/TNFα combined treatments.

**Figure 6:**
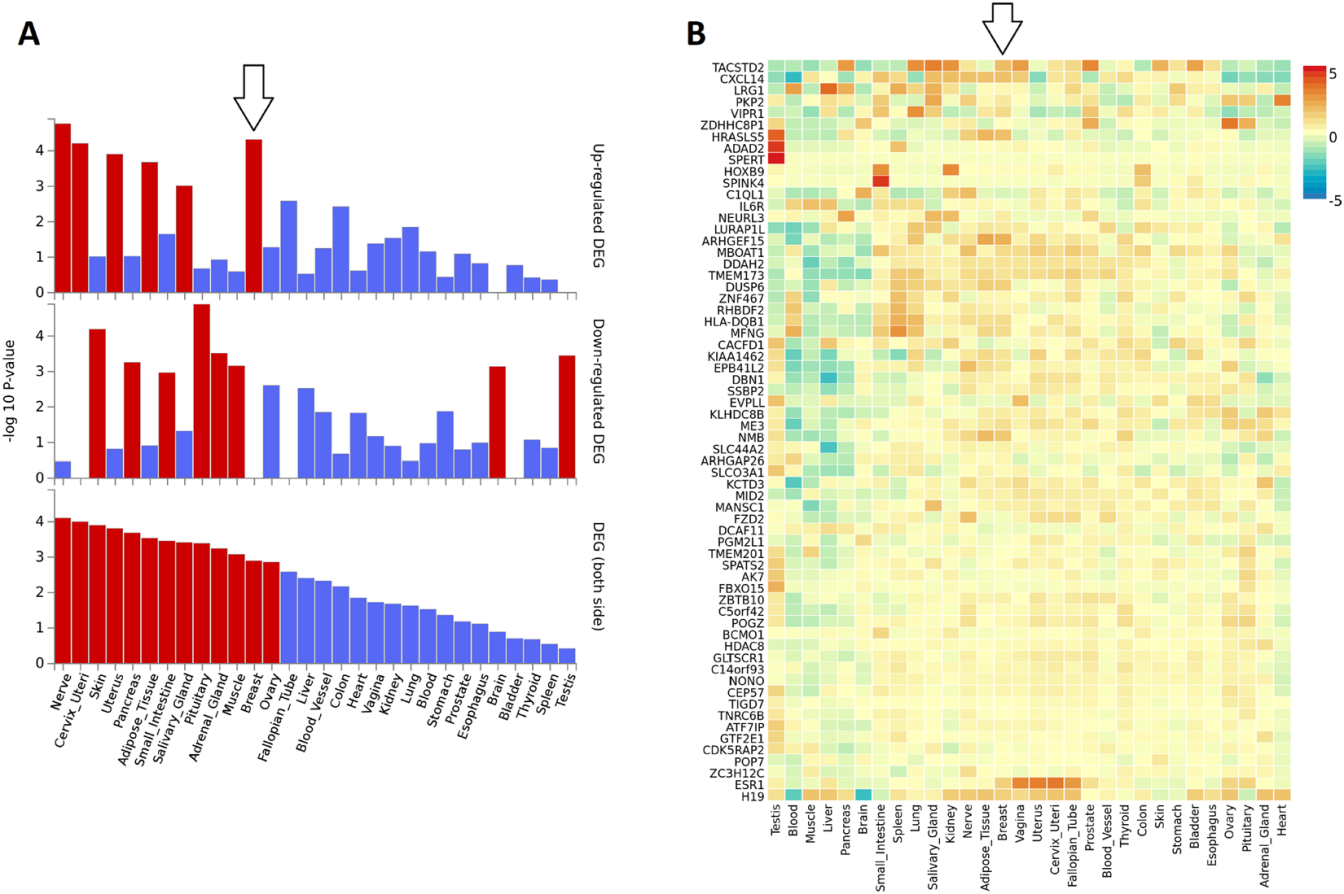
Enrichment in DEG sets analysis of condition-specific differentially expressed genes (CS-DEGs) versus GTEx general tissue types gene sets including breast. Enrichment in DEG sets analysis represent that up-regulation is the main expression pattern of CS-DEGs in normal breast tissue, and down-regulation is a specific consequence of triple treatments with Doxorubicin, Estradiol, and TNFα. (**A**) Enrichment analysis of CS-DEGs versus GTEx general tissue types gene sets. Significantly enriched DEG sets (*Pvalue* ≤ 0.05 and log fold change ≥0.58) are highlighted in red. Expression values show the average of expression of normal tissues based on GTEx database. (**B**) Gene expression heat map of CS-DEGs. Expression values in heatmap show the average of normalized expression per gene (zero mean across samples) in normal tissues based on GTEx (version 6) database.

The topology of the network pathway was constructed using *NASFinder* for the set of context-specific DEGs in response to the combined treatment with Doxorubicin + TNFα  + E2. The integrated network of enriched genes that formed 337 interactions (58 inhibitory and 279 activating) between 186 DEGs included 36 CS-DEGs (Fig. 4) (Supplementary File 3). The node degree distribution of network possessed scale-free topology (*R*^2^ = 0.843), showing the presence of hubs^22^. The centrality of nodes in information flow through the network was assayed and *ESR1* received the high centrality score as the context-specific differentially expressed gene (stress centrality = 6851, betweenness centrality = 0.105), followed by *TP53* and *HIF-1* (Supplementary File 3). Based on the down-regulated pattern of most (43 out of 71) CS-DEGs, and on the down-modulation of the ESR1 transcript itself (fold change in triple treatment = −1.6, p-value < 0.0001), we hypothesized that an interaction with Doxorubicin and TNFα associated responses induced ligand-dependent, ER-mediated repression mechanisms (Fig. 2). A feedback loop whereby ER can repress its own transcription has been previously proposed, and it has been reported that ER can mediate transcriptional repression of target genes, although the mechanisms remain largely elusive^23^. The analysis of the correlation between the fold changes of differentially expressed genes between the double and triple treatments also showed more discrepancy among the down-regulated DEGs (Fig. 5). Multiple linear model regression specified both Doxorubicin + TNFα and Doxorubicin + E2 as significant predictors for Doxorubicin + TNFα  + E2, and analysis of variance model introduced the Doxorubicin + E2 as the main component to fit the model (F-value = 12771, p-value < 0.0001). Next, we analyzed the expression pattern of CS-DEGs in normal breast tissue. Average of normalized expression per gene showed up-regulation as the dominant pattern of expression across genes (Fig. 6A) (Supplementary File 4). Enrichment in DEG sets analysis showed that CS-DEGs in response to the triplet treatment were significantly specific to the normal breast tissue and all enriched genes were overexpressed (up-regulated) (Supplementary File 4) (Fig. 6). These results emphasized the significant association between down-regulated expression pattern of CS-DEGs and combined treatments. Although the experimental protocol that was used introduced the combined treatment at the same time and examined resulting transcriptional changes at steady state, i.e. at a relatively late time point, it is expected that each agent may perturb cell transcriptomics with different kinetics. In particular, E2-mediated responses can be more rapid compared to Doxorubicin-driven p53 activation. Given the enrichment of ER targets among the 71 DEGs that are exclusive to the triple treatment, therefore, these findings suggest the involvement of repression regulatory mechanisms in response to the combinatorial triple treatment.

### Validation of gene expression results in different cell line models

**Figure 7:**
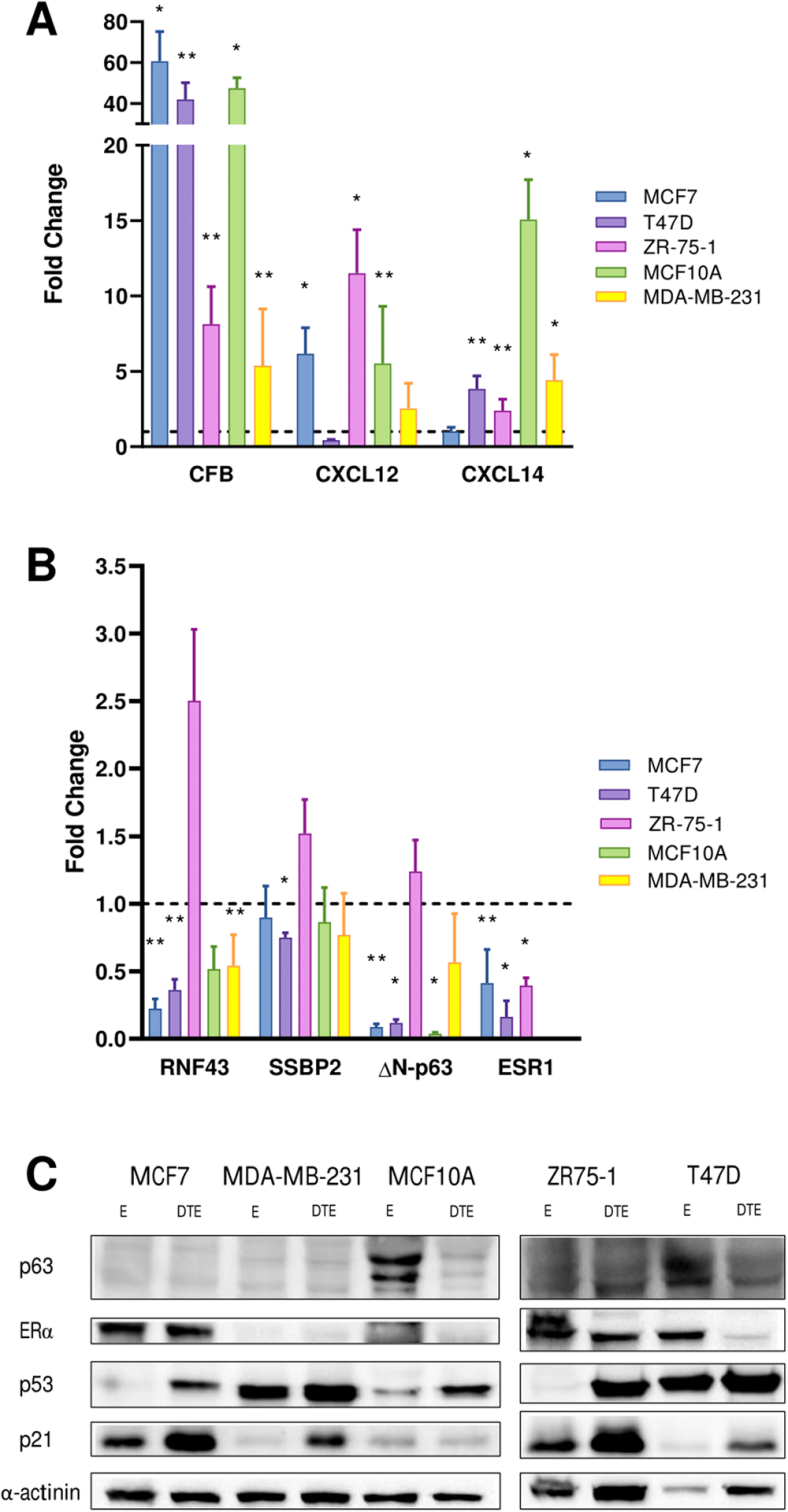
Validation of gene expression data by qPCR and Western Blot. The chosen cell lines differ for p53 and/or ERα status, as described in the Methods section. qPCR results for the indicated genes and the panel of five cell lines, separated by the direction of changes observed in the microarray experiment: (**A**) up-regulated, (**B**) down-regulated. Bars plot the average fold change in response to the Doxorubicin + TNFα + E2 (DTE) treatment compared to the treatment with E2 (set to 1, dashed line). Error bars plot the standard deviation of two biological replicates. (*p < 0.01; **p < 0.05, Student t-test). GAPDH and YWHAZ were used as reference genes. (**C**) Western blot showing the relative expression of p63, ERα p53, p21 in the various cell lines tested and the impact of the Doxorubicin + TNFα + E2 (DTE) treatment. Alpha-Actinin was used as a loading control. The entire blots are presented in Supplementary Fig. 1. Presented is one of two biological replicates that were performed obtaining comparable results. See the Methods section for information on the treatment conditions.

We selected seven genes for validation by qPCR (Fig. 7A,B). Five mammary-gland-derived cell lines differing for p53 and/or ERα status were tested, comparing the Doxorubicin + TNFα  + E2 triple treatment (DTE) with the E2 (E) only treatment. *CFB*, *CXCL12*, and *CXCL14* were chosen as highly up-regulated enriched genes mainly involved in regulation of the immune reactions^24,25^. CFB was significantly up-regulated in all cell lines, hence independently from p53 or ER status. CXCL12 was confirmed as induced by the DTE treatment and particularly so when p53 function is maintained (MCF-7, ZR-75-1 and MCF10A that are p53 wild type, Fig. 7A). Instead we could not confirm the induction of *CXCL14* in MCF-7 although the gene was significantly up-regulated in the other four cell lines, particularly in MCF10A. The down-regulated genes included *RNF43*, *SSBP2*, ΔN-p63, and ESR1 (Fig. 7B). RNF43 was confirmed to be repressed by the triple treatment in MCF-7 and this result was extended to all the other cellular systems with the exception of ZR-75-1. SSBP2 instead was slightly repressed only in T47D. ΔN-p63 was strongly repressed across the board, again with the exception of ZR-75-1. Repression of ER was confirmed in the three ER positive cell lines both at RNA (Fig. 7B) and protein levels (Fig. 7C). However, the reduction is less evident in the highly ERα positive ZR-75-1 cells. Western blot confirmed the stabilization of p53 for the p53 wild type MCF-7, MCF10A, and ZF-75-1 cells in response to the DTE treatment. p21 was induced in all cell lines, although to a much lower extent in the p53 mutant cells, as expected. The repression of ΔN-p63 was confirmed at protein levels in MCF10A and in part also in T47D. The protein was not easily detectable due to low abundance in the other cell lines, consistent with the low relative levels of the endogenous transcript, measured by qPCR (not shown).

## Discussion

We showed that the combinatorial activation of p53, NF-kB and ER transcription factors in MCF-7 induce exclusive gene expression program. We report here that triple treatment of Doxorubicin, TNFα, and E2 resulted in a significant decline in expression of ESR1 in comparison to the single and double treatments. The regulatory network under control of ESR1 showed the implication of its down-regulation on the p53 and immune response pathways. To elucidate the impact of differentially expressed genes in response to the simultaneous activation of p53, NF-kB and ER transcription factors, transcriptomics analysis was done with *NASFinder*, an omics-driven data analysis tool of functional modules used for the analysis of transcriptomics profiles^21^. Functional enrichment analysis showed the differentially regulated genes in response to the activation of p53, NF-kB and ER mainly led to the regulation of network pathways related to the apoptosis, immune response, and basal transcription factors. The validation of *CFB*, *CXCL12*, and *CXCL14* as up-regulated genes in response to the DTE treatment suggests the activation of an inflammatory response, that in the case of CXCL12 could be directly dependent on p53 activation. Among the down-regulated genes we validated *RNF43* and *TP63*. *RNF43* is a negative regulator of WNT signalling^24,25^ and its down-regulation was recently associated with enhanced proliferation and growth^25^. *TP63* is a p53-related transcription factor whose impact can differ depending the relative levels of expression of its isoforms^26^. We confirmed that ΔN-p63 was repressed by the triple treatment in most cell models tested, at least by qPCR. Conversely, TA-p63 did not appear to be modulated by the combination treatment at least in MCF-7 cells (data not shown). This suggests that p63 tumor suppressive functions can be stimulated by the triple treatment and could be involved in the residual induction of p21 expression. We also confirmed the down-regulation of ESR1 in response to the triple treatment both by qPCR and Western blot. Consistently, our transcriptome data revealed that the combination treatments, and particularly the triple treatment Doxorubicin + TNFα + E2 are strongly reducing the output of the E2 treatment in MCF7 cells (Supplementary Fig. 2). The comparison of the regulatory effects of single, double, and triple treatments of compounds identified the context-specific differentially expressed genes. We constructed the integrated regulated network in response to the Doxorubicin + TNFα  + E2 treatment to get additional insight about the enriched exclusive DEGs with network pathways. The results suggested a significant role for *ESR1* as crucial exclusive differentially expressed gene for regulation of *TP53* downstream and ER pathways. Previous studies showed the effect of E2 on p53 cellular localization and cell sensitivity to TNFα^1,11,27^. Treatment of MCF-7 with E2 decreases the transcription factor activity of p53 by transporting it to the cytoplasm, and subsequently, decreases the sensitivity to TNFα induced tumor suppressor effects^28^. In conclusion, these findings suggest that the combination treatment of Doxorubicin, E2 and TNFα may be involved in decreasing the expression and activity of *ESR1*, potentially enhancing the chemotherapy efficacy in ER positive breast cancers. Doxorubicin effects can be modulated by the Estradiol levels which may have implications for using tamoxifen and by the level of immune cells infiltration resulting in TNFα release.

## Methods

### Processing of microarray data

The complete transcriptomics data are accessible through GEO database at NCBI by GSE24065 and GSE50650 accession numbers. The analyses of Doxorubicin/TNFα and Doxorubicin/E2 treatment were reported in^2,17^ and in this study, we focused on the combined treatment of MCF-7 human breast cancer-derived cell line with Doxorubicin, E2 and TNFα in comparison with single or double treatments.

### Identification of differentially expressed genes

We applied Agilent design number 026652 annotation data (chip HsAgilentDesign026652) R object to map between the manufacturer and Gene Symbol Identifiers. DEGs were selected applying a statistical test based on rank products by using RankProd Bioconductor package (pfp < 0.05, absolute log2 fold changes >2)^29^. Every treatment was compared to the mock context.

### Partial least squares discriminant analysis (PLS-DA)

We applied Partial Least Squares Discriminant Analysis (PLS-DA) to cluster the six treatment classes because of its better performance compared to an unsupervised principal component analysis^30^. The classification performance of the PLS-DA model was assessed using 5-fold cross-validation with ten components (repeated 10 times). From the performance results, we chose 6 components to fit the final model.

### Network-based gene set enrichment analysis

**Figure 8:**
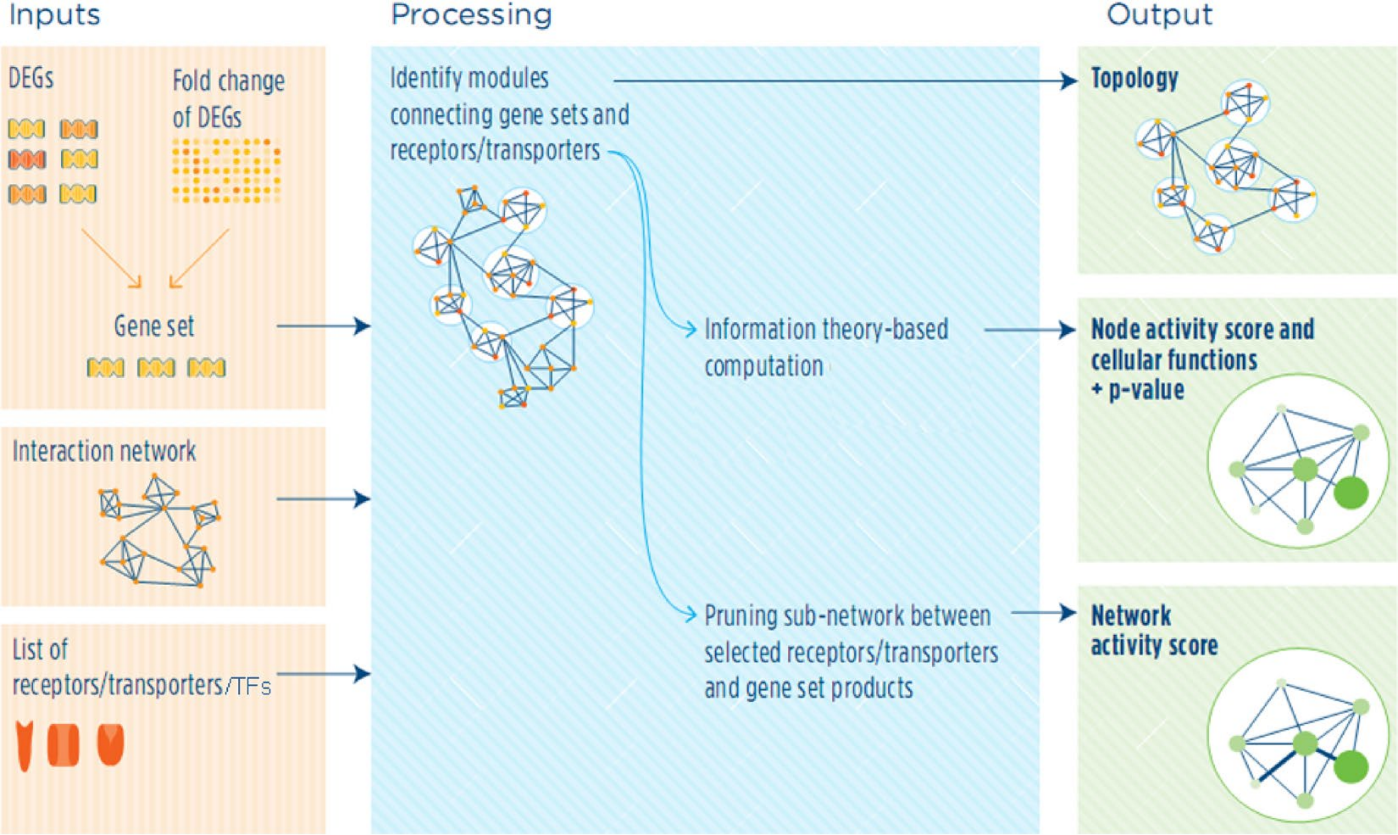
The summarization of network-based gene set enrichment analysis procedure. The main inputs are transcriptomics data, background network, and list of regulators. *NASFinder* is applied to the sets of functionally related genes to identify the biological context (topology) which connects the molecules of interest together and their main regulator (e.g. receptors). Then, the significant sub-networks are used for pathway functional analysis and calculation of network activity score.

We used the COSBI *NASFinder* tool for topological and functional analysis of transcriptomics data^21^. The procedure that *NASFinder* follows to reach the results is as follows (Fig. 8): Inputs: The variable inputs are a list of differentially expressed genes, their fold-difference of expression levels, and list of functionally related gene sets. *NASFinder* uses the topology of a directed and un-weighted interaction network and a list of receptors in this network as constant inputs. *NASFinder* also accesses a repository of interaction datasets for improving the completeness of background network and repository of reference pathways for functional analysis (MSigDB 6.2, all gene sets)^31^. Processing: First, *NASFinder* maps molecules of interest on the background network and then traverses the network from them to receptors (source nodes). It applies an information theory-based method to compute the weight of edges as strength of the relationship of molecules of interest. In the next step, the algorithm identifies the receptors with the highest level of correlation with molecules of interest. As results, it provides a sub-network including shortest paths that connect the molecules of interest to the receptors. Finally, it prunes the sub-network between the selected receptors and molecules of interest and applies it for functional analysis and calculation of network activity score. Outputs: The main outputs are topology that connects the molecules of interest to their main regulator, results of enrichment analysis and network activity score (NAS). NAS describes the impact of differentially regulated network a pathway on the experimental context based on the number of differentially expressed genes in selected reference pathway, mean of their normalized fold change, and the size of selected reference pathway^21^.

### Context-specific differentially expressed genes modules generation and analysis

To extract the regulatory networks from *TP53* and *ESR1* to the specific differentially expressed genes for different combination treatments with Doxorubicin, Estradiol, and TNFα therapeutic agents, we used the *NASFinder* tool^21^. The functional analysis was done based on the 1-neighborhood connectivity network pathway by using the enlarged functional analysis option of *NASFinder*. For visualization of the integrated network, fit the node degree distribution with a power law, and calculation of stress and betweenness centrality scores of nodes, we used the “Network Analyzer” plug-in of Cytoscape^22^. Statistical analyses were conducted and visualized in R. The null hypothesis in the analysis of variance model was no relationship between double and triple treatments and the alternative hypothesis was the relationship between double and triple treatments. Through the F statistics, we considered the strength of pieces of evidence against the null hypothesis.

### Enrichment in DEG sets analysis of CS-DEGs

Tissue specificity is tested using the differentially expressed genes defined for each tissue. *Enrichment in DEG sets analysis* enriches a list of molecules of interest using the differentially expressed gene (DEG) sets defined for each normal tissue. The differentially expressed gene (DEG) sets were selected for 30 general tissue types by performing a two-sided t-test for normalized (zero-mean) expression values (Adjusted P-value ≤ 0.05 and absolute log fold change ≥0.58) from the GTEx database (version 6). For *enrichment in DEG sets analysis* of CS-DEGs, we used all molecules in the topology of pathway networks as the output of *NASFinder*. We used *FUMA* tool for *enrichment in DEG sets analysis* based on publicly available information of 30 normal tissue types in GTEx database^27^.

### Cell culture and treatments

We selected a panel of five different mammary gland-derived cell lines, four obtained from breast cancer patients and one immortalized normal mammary epithelial cell line, MCF10A (a generous gift from Dr. Silvia Soddu, Regina Elena National Cancer Institute, Rome, Italy). MCF7, ZR-75-1, and T47D (Luminal A breast cancer models) were selected as Estrogen Receptor positive (ER+) cells, while MDA-MB-231 (Triple Negative breast cancer model) and MCF10A as ER negative (ER−). MCF7, ZR-75-1, and MCF10A are p53 wild-type cells, while T47D and MDA-MB-231 express mutant p53 (L149F and R280K, respectively). MCF7 were purchased from Interlab Cell Line Collection bank (Genoa, Italy), while ZR-75-1 and MDA-MB-231 were obtained respectively from Dr. Alessio Zippo and Prof. Alessandro Provenzani (Department CIBIO, University of Trento, Italy), while T47D were a gift from Dr. Ulrich Pfeffer (IRCCS Ospedale Policlinico San Martino, Genoa, Italy). MCF7 and MDA-MB-231 were grown in DMEM, whereas ZR-75-1 and T47D in RPMI (Corning, Sigma Aldrich, Milan, Italy). Cell media were supplemented with 10% FBS (Corning), 2 mM L-Glutamine (Corning), and 1X Penicillin/Streptomycin (Corning). In the case of MDA-MB-231 and ZR-75-1, respectively 1% non-essential amino acids (Life Technologies, ThermoFisher Scientific, Milan, Italy) and 1% of Na Pyruvate (Lonza, Milan, Italy) were added. MCF10A were cultured as previously described^28^. Cells were cultured at 37 °C with 5% CO_2_ in a humidified atmosphere. Doxorubicin (MedChemExpress, Monmouth Junction, NJ, USA) was used at the concentration of 1.5 μM. TNFα was from Sigma Aldrich and used at the concentration of 5 ng/ml for MCF7 and MCF10A cells and at 10 ng/ml for T47D, ZR-75-1 and MDA-MB-231 that were shown to be less responsive to this cytokine. 17-β Estradiol (E2, Sigma Aldrich) was used at the concentration of 1 nM. Cells were harvested 16 hours after the administration of the drugs. In previous experiments, we had shown similar results with both a 10-hour and a 16-hour treatment schedule^2,28^.

### RNA isolation and RT-qPCR

Total RNA was extracted using RNeasy® Mini Kit (Qiagen, Milan, Iatly), converted into cDNA with PrimeScript^TM^ RT reagent Kit (Takara, Diatech Lab Line, Ancona, Italy) and RT-qPCR was performed with 25 ng of template cDNA in 384 well-plate (BioRad, Milan, Italy) using qPCRBIO SyGreen 2X Mix (PCR Biosystems, Resnova, Ancona, Italy) with the CFX384 Real-Time detection system (BioRad). YWHAZ and GAPDH were used as reference genes to obtain the relative fold change by the ΔΔCt method as previously described^29^. Primers were designed using Primer-BLAST online tool (https://www.ncbi.nlm.nih.gov/tools/primer-blast/), checked for specificity and efficiency. qPCR primer sequences are available upon request.

### Western blot

Total protein extracts were obtained by lysing the cells using RIPA buffer supplemented with protease inhibitors (Roche, Milan, Italy) and the proteins were quantified by the BCA method (Pierce, ThermoFisher Scientific); 30–50 μg of proteins were loaded on 12% polyacrylamide gels and SDS-PAGE was performed, transferring proteins on HyClone nitrocellulose membranes (Amersham, Euroclone, Milan, Italy) that were probed over-night at 4 °C with specific antibodies diluted in 1% skimmed milk-PBS-0.1% Tween solution: ERα (A300-4984, Bethyl Laboratories, Tema Ricerca, Bologna, Italy), p53 (DO-I, sc-126, Santa Cruz Biotechnologies, Milan, Italy), p21 (EPR362, ab109520, Abcam, Prodotti Gianni, Milan, Italy), p63 (4A4, sc-8431, Santa Cruz Biotechnologies), α-actinin (H2, sc-17829, Santa Cruz Biotechnologies). Detection was performed by ECL (GE Healthcare, Euroclone) using ChemiDoc XRS+ (BioRad) imaging system.

## Supplementary information

Supplementary Figures

Supplementary Information title page

Dataset 1

Dataset 2

Dataset 3

Dataset 4
