# Supplementary material for: Regulatory Crosstalk of Doxorubicin, Estradiol and TNFα Combined Treatment in Breast Cancer-derived Cell Lines
Source: Sci Rep. 2019 Oct 23;9:15172. doi: 10.1038/s41598-019-51349-9 (PMC6811586; doi:10.1038/s41598-019-51349-9)
Supplement: Supplementary file 1 — Supplementary Figures [file 41598_2019_51349_MOESM1_ESM.docx]

**Supplementary Figures:**

**Regulatory Crosstalk of Doxorubicin, Estradiol and TNFα Combined Treatment in Breast Cancer-derived Cell Lines**

Isar Nassiri^1,2^, Alberto Inga^3^, Erna Marija Meškytė^4,5^, Federica Alessandrini^4^, Yari Ciribilli^4^, and Corrado Priami^2,6*^

^1^Department of Oncology, Weatherall Institute for Molecular Medicine, University of Oxford, UK

^2^The Microsoft Research – University of Trento Centre for Computational and Systems Biology (COSBI), Rovereto (TN), Italy

^3^Laboratory of Transcriptional Networks, Department CIBIO, University of Trento, 38123 Trento, Italy

^4^Laboratory of Molecular Cancer Genetics, Department CIBIO, University of Trento, 38123, Trento, Italy

^5^Department of Biological Models, Life Sciences Centre, Institute of Biochemistry, Vilnius University, Lithuania

^6^Dipartimento di Informatica, Università di Pisa, Pisa, Italy

*Correspondence and requests for materials should be addressed to C.P. (priami@cosbi.eu)

**
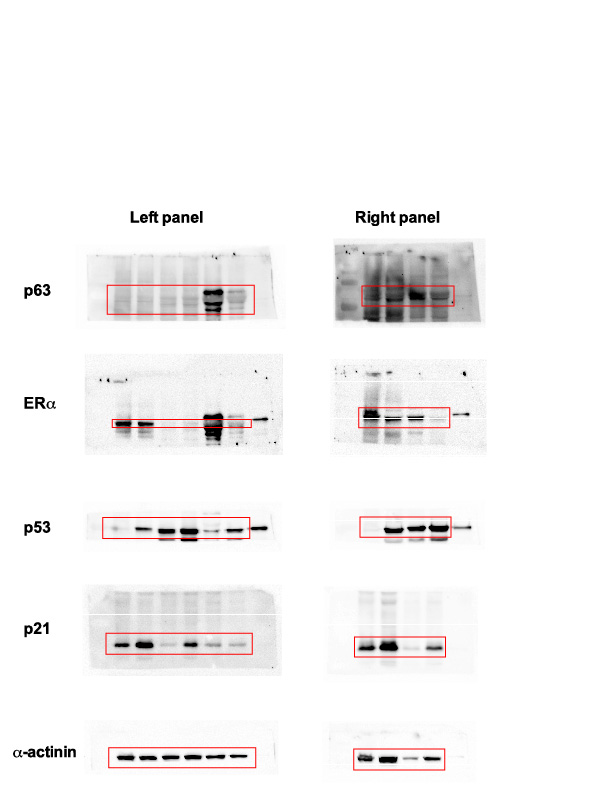
**

**Supplementary Figure 1**. Uncropped and full-length gels of a Western blot showing the relative expression of p63, ERα p53, p21 proteins in the various cell lines tested and the impact of the Doxorubicin + TNFα + E2 (DTE) treatment. Alpha-Actinin was used as the loading control. The red boxes indicate the WB panels presented in Figure 7.

**Supplementary Figure 2.** Gene-specific as well as the global impact of combination treatments on the impact of E2 treatment in MCF7 cells. **A**) The upregulated differentially expressed gene list from MCF7 cells treated with 1nM estradiol (E2) (Supplementary File 1) were interrogated using Enrichr web tool and 37 direct ER transcriptional targets were identified based on published ChIP-seq data obtained in the same cell line. The log_2_ fold changes for these genes are presented in the form of a heatmap for the indicated double and triple treatments. **B**) Heatmap view for all differentially expressed genes in our dataset; individual biological replicates are plotted and clustered separately. **C**) Heatmap view of the impact of double and triple treatments compared to E2 single treatment for genes identified by *NASFinder* as part of the direct ERα topology network. See also Supplementary File 3 for information on the type of interactions.
